# Welcome to *Tropical Medicine and Infectious Disease*—A New Era in Open Access Publication

**DOI:** 10.3390/tropicalmed1010001

**Published:** 2016-06-14

**Authors:** Peter A. Leggat, John Frean

**Affiliations:** 1 College of Public Health, Medical and Veterinary Sciences, James Cook University, Townsville, Queensland 4811, Australia; 2 Centre for Opportunistic, Tropical and Hospital Infections, National Institute for Communicable Diseases, and University of the Witwatersrand, Johannesburg 2131, South Africa

Historically, tropical medicine emerged from a multidisciplinary background as a result of progress in the areas of public health and hygiene, travel and exploration, biology and evolution, and the germ theory of disease [1]. The *World Health Organization* (WHO) defines tropical diseases as all diseases that occur solely, or principally, in the tropics’ [2]. WHO’s focus has been on a range of infectious and vector-borne diseases, such as malaria, leishmaniasis, schistosomiasis, onchocerciasis, lymphatic filariasis, Chagas’ disease, African trypanosomiasis, and dengue. The recent or current Ebola, Zika, chikungunya, and yellow fever outbreaks illustrate how tropical diseases can re-emerge or expand their ranges, and cause havoc. However, there has been an epidemiological transition in many countries in many socio-economic and health factors, which has included an increasing burden of chronic and degenerative diseases. Tropical diseases have the potential to impact on tourists, business travellers, expatriate workers, migrants, and refugees in endemic regions, and travel can also facilitate the spread of infectious diseases globally.

The Australasian College of Tropical Medicine has partnered with long term electronic publisher, MDPI, to develop an international, scientific, open access journal of tropical medicine and infectious disease to be published quarterly online. We have named this new journal, *Tropical Medicine and Infectious Disease*. It has been endorsed as the Official Journal of The Australasian College of Tropical Medicine (ACTM) and its joint Faculties of Travel Medicine and Expedition and Wilderness Medicine. Both MDPI and the ACTM are celebrating anniversaries this year, being 20 and 25 years old respectively, and are both proud of their respective traditions in the world of publishing. 

MDPI publishes 150 peer reviewed, scientific, open access, electronic journals in a broad range of fields. It has offices in four cities, being headquartered in Basel, Switzerland and having additional offices in China and Spain. MDPI is a member of all the major professional publisher associations, including OASPA, STM, or COPE and has a strong track record in building the indexing and abstracting support for each of its journals [3]. Part of the strategic plan for the new journal in the short to medium term is to build the coverage by leading indexing and abstracting databases. MDPI also offers the flexibility to develop Special Issues under the leadership of one or more guest editors, which could have the attraction and added spinoff as being published as online books.

The ACTM is particularly excited about this project being launched in its 25th Anniversary Year. The ACTM itself has been publishing the *Annals of the ACTM* since 1995, initially as conference proceedings and then as a peer-reviewed journal from 2002. All of these are available online [4]. The College has also published various directories, textbooks and newsletters over the years. Some of these are available electronically, including our popular *Dictionary of Tropical Medicine* [5]. The College has been very supportive of open access for its publications, wherever possible. 

The aim of *Tropical Medicine and Infectious Disease* is to publish authoritative and original articles, critical and systematic reviews, editorials, perspectives, short communications, commentaries, book reviews, letters to the editor and special issues on all aspects of tropical medicine and tropical infectious disease. All articles will be submitted through MDPI’s excellent online journal submission platform and are subject to editorial and peer-review. The MPDI Editorial Process is extremely rigorous [6], but it also aims to produce timely outcomes by involving its dedicated supporting teams. Once an article is accepted, the manuscript immediately enters the publication cycle to be published online within 8–10 days.

*Tropical Medicine and Infectious Disease* will publish on all tropical diseases of global significance, as well as neglected tropical diseases, as defined from time-to-time by the WHO. The scope of the journal includes, but is not limited to clinical tropical medicine, tropical infectious diseases, parasitology and entomology, bacteriology, mycology and virology, epidemiological and social science studies, chemotherapy and pharmacology, immunology, disease prevention, control and elimination, emerging and re-emerging infectious diseases, emerging public health threats, and global health and One Health.

We are very proud to welcome our foundation international Editorial Board, an extremely talented group of more than 20 senior academics and clinicians from six continents with global outreach. We also warmly welcome our Assistant Editor, Anna Liu, who has championed the development of the journal and will be a key contact for both contributors and Editorial Board members. The team will be led by our Editor-in-Chief, John Frean from South Africa, and our Deputy Editor-in-Chief, Peter A. Leggat, AM, from Australia. We will be reviewing and building our Editorial Board and reviewing teams in the coming years and the Editor-in-Chief would welcome nominations from interested leaders in the fields of tropical medicine and infectious disease. We are also looking forward to receiving your research, reviews and other scholarly contributions and we welcome suggestions or ideas that can enhance this new journal.

On behalf of the Editorial Board, we invite you to submit your manuscripts and suggestions for Special Issues and guest editorials to *Tropical Medicine and Infectious Disease*. 
